# Supplementary material for: Identification and Characterization of Colletotrichum Species Causing Sorghum Anthracnose in Kenya and Screening of Sorghum Germplasm for Resistance to Anthracnose
Source: J Fungi (Basel). 2023 Jan 11;9(1):100. doi: 10.3390/jof9010100 (PMC9864066; doi:10.3390/jof9010100)
Supplement: Supplementary file 1 [file jof-09-00100-s001.zip › jof-2060908-supplementary.pdf]

Journal of Fungi

MDPI

Supplementary Material

Supplementary Tables

Supplementary Table S1. List of samples with their respective symptoms observed in the field.

County	S/No	Sample ID	Symptoms Observed in the Field
Kitui	1	KT001-A	Small and elongated elliptical necrotic lesions on the leaf blade
	2	MUT006-B	Small, elliptical red lesions with tan centers<br>Presence of fruiting bodies on dark lesions
	3	IKA009-G	Small and elongated elliptical necrotic lesions on the leaf blade
	4	KY006	Small, elliptical red lesions with tan centers<br>Presence of fruiting bodies on dark lesions
	5	WA022	Small, elliptical red lesions with tan centers<br>Presence of fruiting bodies on dark lesions
	6	KAY062	Small, elliptical red lesions with tan centers<br>Reddish discoloration on the midrib
	7	IKU027	Small, elliptical red lesions with tan centers<br>Reddish discoloration on the midrib
	8	IKT011	Dark brown circular spots<br>Necrotic sunken lesions on the leaf surface
	9	KY049	Both small and elongated elliptical necrotic lesions on the leaf blade<br>Red lesions on the midrib and leaf sheath
	10	KAM035	Small and elongated elliptical necrotic lesions on the leaf blade<br>Necrotic sunken lesions on the leaf blade
Makueni	11	KAT015-A	Both small and elongated elliptical necrotic lesions on the leaf blade
	12	MAK007-D	Dark brown circular spots<br>Necrotic sunken lesions on the leaf surface
	13	W008-L	Small and elongated elliptical necrotic lesions on the leaf blade
	14	MAV071	Dark brown circular spots<br>Necrotic sunken lesions on the leaf surface
	15	KAT055	Small and elongated elliptical necrotic lesions on the leaf blade<br>Necrotic sunken lesions on the leaf blade
	16	KAT050	Small and elongated elliptical necrotic lesions on the leaf blade<br>Necrotic sunken lesions on the leaf blade
	17	KK038	Both small and elongated elliptical necrotic lesions on the leaf blade
Machakos	18	MTH022-A	Small and elongated elliptical necrotic lesions on the leaf blade<br>Necrotic sunken lesions on the leaf blade
	19	KI080	Small and elongated elliptical necrotic lesions on the leaf blade<br>Necrotic sunken lesions on the leaf blade
	20	MKS078	Red lesions on the midrib and leaf sheath
	21	MV089	Small, elliptical red lesions with tan centers<br>Presence of fruiting bodies on dark lesions
	22	MAT094	Small, elliptical red lesions with tan centers<br>Reddish discoloration on the midrib
	23	ND098	Small, elliptical red lesions with tan centers<br>Reddish discoloration on the midrib
	24	MTH092	Small, elliptical red lesions with tan centers<br>Reddish discoloration on the midrib
	25	MAS101	Dark brown circular spots<br>Necrotic sunken lesions on the leaf surface

	26	MAS103	Dark brown circular spots<br>Necrotic sunken lesions on the leaf surface

Supplementary Table S2. Results of pathogenicity tests at 10 d after inoculation of the sorghum cultivars expressed as a disease severity score (mean ± standard deviation). Severity score 0 –no symptoms, 1 – discrete lesions (necroses) less than 2 mm in length, 2 – lesion occupying less than 25 % of leaf surface, 3 – lesion occupying half of leaf surface, 4 – lesion occupying entire leaf surface and 5 –lesion with abundant sporulation .

County	Isolate ID	Pathogenicity
Makueni	KAT015-A	3.7±1.3
Kitui	KAT001-A	4.3±0.9
Makueni	W008-L	4.0±1.4
Kitui	MUT006-B	3.9±1.4
Makueni	MAK017-D	3.6±1.3
Kitui	IKA009-G	3.8±1.3
Machakos	MTH022-A	4.1±1.1

Supplementary Table S3. Comparison between *Colletotrichum sublineola* and *Colletotrichum eremochloae*. Source; Crouch and Tomaso (2021).

***Colletotrichum sublineola* (Strain MB529591)**	***Colletotrichum eremochloae* (Strain CBS129661)**
**Colony color:** Ranges from white to pink and dark gray to clear-gray<br>**Conidia:** Falcate, oval to fusiform, hyaline, unicellar, non-septate<br>with rounded ends.<br>**Hyphal appressoria:** abundant in culture Single-celled, dark to hyaline, globose to sub-globose shaped<br>**Nucleotide sequences;** Has a fixed nucleotide differences at nuclear internal transcribed spacer region Apn2, Apn2/Mat1 and Sod2 when amplified and sequenced with PCR primers ITS4/ITS5, Mat1M72F/ Mat1M72R, Apn1W1F/Apn1W1R and SOD625F/SOD625R.	**Colony color:** light to dark grey without visible conidial masses with a bright yellow perimeter surrounding the colony<br>**Conidia:** one-celled, Falcate sometimes fusiform, Smooth, hyaline, Setae & Sclerotia absent<br>**Hyphal appressoria:** abundant in culture, medium to dark brown, globose, ovoid, Clavate, lobate or multilobate, and obtuse. apices<br>**Nucleotide sequences;** Has a fixed nucleotide differences at nuclear internal transcribed spacer region Apn2, Apn2/Mat1 and Sod2 when amplified and sequenced with PCR primers ITS4/ITS5, Mat1M72F/ Mat1M72R, Apn1W1F/Apn1W1R and SOD625F/SOD625R.

Supplementary Table S4. Basic information of the fungal strains used to construct the phylogenetic tree.

ID	Accession number	*Species*	Country	year	Host
IKA009-G*		*C. sublineola*	Kenya		Sorghum sp.
KAT015-A*		*C. sublineola*	Kenya		Sorghum sp.
KT001-A*		*C. sublineola*	Kenya		Sorghum sp.
MAK017-D*		*C. sublineola*	Kenya		Sorghum sp.

MTH022-A*		*C. sublineola*	Kenya		Sorghum sp.
MUT006-B*		*C. sublineola*	Kenya		Sorghum sp.
W008-L*		*C. sublineola*	Kenya		Sorghum sp.
IR-06	AB242422	*C. falcatum*	India	N/A	Sorghum sp.
MAFF 511474	AB439813	*C. sublineola*	Japan	2000	Sorghum bicolor
MAFF 243051	AB738855	*C. hsienjenchang*	Japan	2011	Phyllostachys bambusoides
S26	EF016297	*C. truncatum*	China	2006	Tobacco
S35	EF016299	*C. truncatum*	China	2006	Tobacco
DAOM116226	EU400155	*C. brassicae*	Canada	2008	N/A
1818	FJ236462	*C. dematium*	Bulgaria	N/A	Statice
CY125	HQ607974	*C. caudatum*	USA	2006	Cyphomyrmexwheeleri nest
Cf-17	HQ833662	*C. falcatum*	N/A	N/A	N/A
CBS 129663	JQ005774	*C. cereale*	N/A	N/A	N/A
CDLG3	JQ400004	*C. liriopes*	China	N/A	Hemerocallis fulva
Co-38-Zac	JQ658887	*C. sublineola*	Mexico	N/A	Sorghum halepense
CDRJ01	JQ677042	*C. dematium*	South Korea	N/A	Rohdea japonica Roth
I137	KC598120	*C. spaethianum*	Brazil	N/A	Hemerocallis lilioasphodelus
MAFF305403	KC790948	*C. paspali*	India	N/A	N/A
MAFF305404	KC790949	*C. hanaui*	India	N/A	N/A
MAFF511115	KC790951	*C. nicholsonii*	India	N/A	N/A
MAFF511155	KC790952	*C. eleusines*	India	N/A	N/A
MTCC10326	KC790960	*C. navitas*	India	N/A	N/A
SimpsonStem1	KU171107	*C. sublineola*	USA	N/A	Sorghum bicolor
CSB_F052	KU574683	*C. echinochloae*	Kenya	N/A	Brachiaria sp.
VIC32T	KU821158	*C. coccodes*	Australia	N/A	potato
USANY	KU821176	*C. nigrum*	USA	N/A	potato
BRIP 63314c	KX069822	*C. tofieldiae*	Australia	N/A	Grevillea crithmifolia
RJ6	MH005034	*C. liriopes*	USA	N/A	Rohdea japonica
I-3	MN636355	*C. falcatum*	Bangladesh	N/A	Sugarcane
CZPMP-17	MN889462	*C. sublineola*	India	2019	Pennisetum
SI24	MT077154	*C. duyunense*	China	N/A	Sinoseneciooldhamianus
CNUCC 303221	MT199610	*C. bambusicola*	China	N/A	Phyllostachys aureosulcata
CNUCC 307304	MT199631	*C. guangxiense*	China	N/A	Phyllostachys edulis
MV101	MW054252	*C. graminicola*	N/A	N/A	N/A
YMF 1.06700	MW722955.1	*C. garzense*	China	N/A	N/A
IMI 279189	NR_171193.1	*C. axonopodis*	Australia	N/A	Axonopusfissifolius
CBS 129664	MH865530.1	*C. eremochloae*	N/A	N/A	N/A
CBS 129661	MH865527.1	*C. eremochloae*	N/A	N/A	N/A

* Represents strains from the current study.

Supplementary Table S5. Regression analysis between sorghum anthracnose and yield components. *Dependent Variable: Anthracnose .

Coefficients*					
	Unstandardized Coefficients		Standardized Coefficients		
Model	B	Std. Error	Beta	t	Sig.
(Constant)	6.207	13.729		.452	.665
Grain weight	.139	.139	1.185	1.001	.350
Grain Yield t/ha	-3.396	4.653	-2.182	-.730	.489
100seeds (g)	-7.249	3.280	-1.745	-2.210	.063

Supplementary Figures

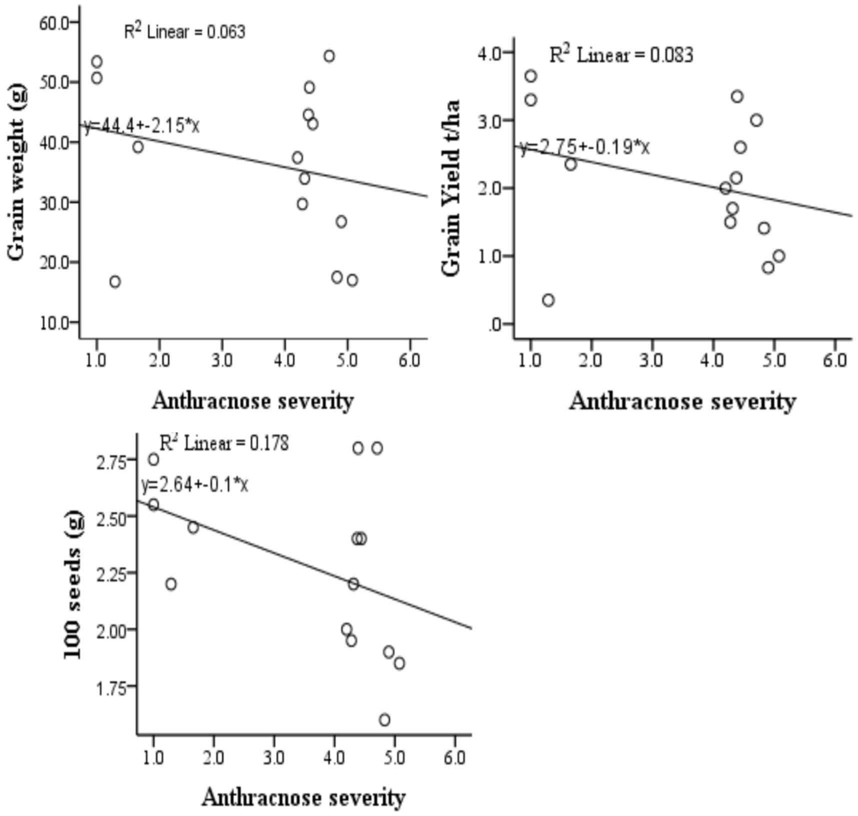

Supplementary Figure **S1.** Regression analysis on the effect of sorghum anthracnose on yield of local and improved genotypes obtained from lower eastern Kenya.

References

1. Crouch, J.A.; Tomaso, PM. Anthracnose disease of centipegrass turf caused by *Colletotrichum eremochloae*, a fungal species closely related to Colletotrichum sublineola. *Mycologia* **2021**, *104*, 1085–1096.
